# Hemoadsorption therapy for myoglobin removal in rhabdomyolysis: consensus of the hemoadsorption in rhabdomyolysis task force

**DOI:** 10.1186/s12882-024-03679-8

**Published:** 2024-07-31

**Authors:** Lui Forni, Filippo Aucella, Gabriella Bottari, Stefan Büttner, Vincenzo Cantaluppi, Dietmar Fries, Jan Kielstein, Detlef Kindgen-Milles, Claus Krenn, Andreas Kribben, Andreas Meiser, Steffen Mitzner, Marlies Ostermann, Vedran Premuzic, Caroline Rolfes, Christina Scharf, Stefan Schunk, Zsolt Molnar, Alexander Zarbock

**Affiliations:** 1 Critical Care Unit, Royal Surrey Hospital, Guildford, Surrey UK; 2 School of Medicine, University of Surrey, Kate Granger Building, Guildford, UK; 3 “Casa Sollievo della Sofferenza” Foundation, Scientific Institut for Research and Health Care, Viale Cappuccini, 1, San Giovanni Rotondo (FG), 71013 Italy; 4 Pediatric Intensive Care Unit, Bambino Gesu Children’s Hospital, IRCCS, Rome, 00165 Italy; 5 Cardiology, Pulmonology, Nephrology and Intensive Care Medicine, Klinikum Aschaffenburg- Alzenau, Academic Teaching Hospital of Julius-Maximilians-University Würzburg, Aschaffenburg, Germany; 6 Nephrology and Kidney Transplantation Unit, Department of Translational Medicine, University of Piemonte Orientale (UPO), Novara, Italy; 7 “Maggiore della Carità” University Hospital, via Gen. P. Solaroli 17, Novara, 28100 Italy; 8 Department for Anaesthesia and Critical Care Medicine, Medical University, Christoph-Probst- Platz 1, Innrain 52 A, Fritz-Pregl-Straße 3, Innsbruck, 6020 Austria; 9 Medical Clinic V, Nephrology, Rheumatology, Blood Purification, Academic Teaching Hospital Braunschweig, Naumburgstraße 15, D-38124 Braunschweig, Germany; 10 Department of Anesthesiology, University Hospital Duesseldorf, Heinrich-Heine University, Moorenstr.5, D-40225 Duesseldorf, Germany; 11 Clinic for Anaesthesia, General Intensive Care Medicine and Pain Therapy, Medical University of Vienna, Wahringer Gurtel 18-20, Vienna, 1090 Austria; 12 Universitätsklinikum Essen (AöR) Nephrology Clinic, Medizinisches Zentrum, 2.104 Hufelandstraße 55, D-45147 Essen, Germany; 13 Department of Anesthesiology, Intensive Care Medicine and Pain Therapy, Saarland University Hospital, D-66424 Homburg, Germany; 14 Fraunhofer IZI Rostock, Schillingallee 68, 18057 Rostock, Germany; 15 Department of Critical Care, King’s College London, Guy’s & St. Thomas’ Hospital, London, SE1 9RT UK; 16 Department for Nephrology, Hypertension, Dialysis and Transplantation, School of Medicine, UHC Zagreb Croatia, University of Zagreb, Šalata ul. 2, Zagreb, 10000 Croatia; 17 Department for Anesthesiology, Intensive Care Medicine, Pain Therapy and Emergency Medicine, GNH Klinikum Kassel, Mönchebergstraße 41-43, D-34125 Kassel, Germany; 18 Department of Anesthesiology, LMU University Hospital, LMU Munich, Geschwister-Scholl- Platz 1, D-80539 München, Germany; 19 Department of Internal Medicine 4, Nephrology and Hypertension, Saarland University Hospital, Kirrberger Str. 100, D-66421 Homburg/Saar, Germany; 20 Department of Anesthesiology and Intensive Therapy, Semmelweis University, Üllői út 78, Budapest, H-1082 Hungary; 21 Department of Anaesthesiology and Intensive Therapy, Poznan University of Medical Sciences, Collegium Maius, Fredry 10, Poznan, 61-701 Poland; 22 CytoSorbents Europe, Müggelseedamm 131, D-12587 Berlin, Germany; 23 Department of Anesthesiology, Intensive Care and Pain Medicine, University Hospital Münster, Albert-Schweitzer-Campus 1, D-48149 Münster, Germany

**Keywords:** Rhabdomyolysis, Acute kidney injury, Renal replacement therapy, Blood purification, Hemoadsorption, CytoSorb

## Abstract

**Background:**

Rhabdomyolysis describes a syndrome characterized by muscle necrosis and the subsequent release of creatine kinase and myoglobin into the circulation. Myoglobin elimination with extracorporeal hemoadsorption has been shown to effectively remove myoglobin from the circulation. Our aim was to provide best practice consensus statements developed by the Hemoadsorption in Rhabdomyolysis Task Force (HRTF) regarding the use of hemadsorption for myoglobin elimination.

**Methods:**

A systematic literature search was performed until 11th of January 2023, after which the Rhabdomyolysis RTF was assembled comprising international experts from 6 European countries. Online conferences were held between 18th April − 4th September 2023, during which 37 consensus questions were formulated and using the Delphi process, HRTF members voted online on an anonymised platform. In cases of 75 to 90% agreement a second round of voting was performed.

**Results:**

Using the Delphi process on the 37 questions, strong consensus (> 90% agreement) was achieved in 12, consensus (75 to 90% agreement) in 10, majority (50 to 74%) agreement in 13 and no consensus (< 50% agreement) in 2 cases. The HRTF formulated the following recommendations: (1) Myoglobin contributes to the development of acute kidney injury; (2) Patients with myoglobin levels of > 10,000 ng/ml should be considered for extracorporeal myoglobin removal by hemoadsorption; (3) Hemoadsorption should ideally be started within 24 h of admission; (4) If myoglobin cannot be measured then hemoadsorption may be indicated based on clinical picture and creatinine kinase levels; (5) Cartridges should be replaced every 8–12 h until myoglobin levels < 10,000 ng/ml; (6) In patients with acute kidney injury, hemoadsorption can be discontinued before dialysis is terminated and should be maintained until the myoglobin concentration values are consistently < 5000 ng/ml.

**Conclusions:**

The current consensus of the HRTF support that adjuvant hemoadsorption therapy in severe rhabdomyolysis is both feasible and safe and may be an effective method to reduce elevated circulating levels of myoglobin.

## Introduction

Rhabdomyolysis was described by Bywaters and Beall in the British Medical Journal (BMJ) in 1941 who demonstrated the presence of pigmented casts in the renal tubules of victims of bombing during World War 2 [1]. Of note, this syndrome had previously been described by German pathologists and a published summary of the literature provided a description that tallied exactly with that in the BMJ [2]. Often described as “crush syndrome” [3], rhabdomyolysis refers to the breakdown of skeletal muscles following severe injury which may complicate trauma, major surgery, high voltage electrocution, intoxications, drug abuse and infection [4]. It is best viewed as a multifactorial syndrome, the clinical presentation of which is variable and can range from mild muscle pain, weakness, and confusion [5] through to critical illness requiring multi-organ support [4, 6]. Myoglobin release plays a pivotal role in the pathophysiological mechanisms behind rhabdomyolysis related organ dysfunction particularly acute kidney injury (AKI). Following injury, serum myoglobin concentrations rise, but due to rapid renal clearance, circulating levels may decrease within the first 24 h after the onset of symptoms [7]. However, when the myoglobin concentration exceeds the reabsorbing capacity of the kidney, myoglobulin appears in the urine (myoglobinuria) and when detected in the first 24 h after an injury, is pathognomonic for the diagnosis of rhabdomyolysis [8]. Although there is no current consensus definition for rhabdomyolysis, a recent systematic review recommends defining rhabdomyolysis as a clinical syndrome of acute muscle weakness, myalgia, and muscle swelling combined with a creatinine kinase (CK) cut-off value of > 1000 IU/L or a 5-fold increase above the upper limit of the local laboratory reported normal range (for mild rhabdomyolysis). Additionally, measured myoglobinuria and AKI might serve as indicators for the severity of rhabdomyolysis [9]. An estimated 10 − 40% of patients with rhabdomyolysis develop AKI, with a risk that increases not only with the degree of CK rise, but also in the presence of volume depletion, sepsis and acidosis and the development of AKI increases the observed in-hospital mortality significantly [10]. Given the fundamental role of myoglobin in the development of the rhabdomyolysis syndrome it follows that early and effective reductions in elevated levels may prevent the development of AKI and its further consequences, or at least shorten its duration.

Currently therapeutic interventions for the management of rhabdomyolysis and associated AKI mostly focus on fluid therapy, urine alkalinisation and diuretic support to improve both tubular flow and pH with attempts to reduce myoglobin precipitation [11]. However, an alternative approach is the removal of circulating myoglobin through extracorporeal techniques and renal replacement therapy (RRT) thereby enhancing myoglobin clearance. Although these techniques have been considered for the treatment of rhabdomyolysis, there is insufficient evidence to support such an approach over conservative therapies to-date [12]. Indeed, RRT tends to be deemed as necessary only when AKI is established or acute indications for RRT develop such as significant hyperkalemia [11]. The molecular weight of myoglobin is 17 kDa, and as such high flux, high permeability membrane hemofiltration or hemodiafiltration are potentially suited for the removal of myoglobin [13]. Moreover, myoglobin removal has been reported by means of high-permeability dialysis [14] and high-flux hemofiltration [15]. The use of newer hyper-permeable dialyzers (high cut-off and medium cut-off dialyzers) also provide myoglobin clearance [16], with the clearance with high cut-off dialyzers being superior to high-flux dialyzers [17]. However, their use has also been associated with loss of albumin [18] and coagulation factors [19]. An alternative approach towards myoglobin clearance is the use of polymer-based adsorption technologies such as CytoSorb^®^, which consists of porous beads with adsorption capability especially for hydrophobic substances within a range of up to 60 kDa, including cytokines, bilirubin and myoglobin [20–22]. The potential for the adsorption of both myoglobin and cytokines offers the possibility of targeting two well-described contributors to the underlying pathophysiology of rhabdomyolysis and associated AKI. Myoglobin removal has been demonstrated where the percent reduction in plasma myoglobin levels during one passage through a hemoadsorption device was measured. The percent reduction was 80% initially, rapidly declining to 40%, 20% 15% and 12% after 30 min, 2, 4 and 8 h, respectively [23]. Clearance is then determined as a product of plasma flow multiplied by percent reduction of myoglobin which, in turn, depends on its plasma concentration. Interestingly, the saturation kinetics of the hemoadsorber is not myoglobin concentration dependant and may reflect competitive binding to the adsorption sites on the beads inside adsorber from other molecules. Therefore, the efficacy of myoglobin elimination will decrease rapidly over time and the cartridge should be exchanged after 8 to 12 h where further myoglobin elimination seems indicated. Myoglobin elimination could avert permanent kidney damage by avoiding its deposition on the kidney [24], while simultaneous removal of excessive cytokine levels represents an option in patients with rhabdomyolysis and concomitant sepsis or other inflammatory states, enabling achievement of two potential treatment goals [25].

To-date the literature reveals a total of 15 clinical studies on the use of CytoSorb^®^ hemoadsorption in rhabdomyolysis [23–37]. The published data confirms that the use of hemoadsorption is associated with a reduction in myoglobin and other markers of inflammation. However, in the absence of robust trial data recommendations as to best clinical practice are difficult hence a consensus conference was convened to examine the evidence and provide recommendations based on a modified Delphi process.

## Methods

### Objectives

The aim of this initiative was to summarize and evaluate the available data, define current practice, identify knowledge gaps and research questions for the future and finally to give recommendations based on a consensus of 19 international experts in the field as members of the Task Force. During this Delphi process we considered application of hemoadsorption as either as a stand-alone therapy or combined with continuous renal replacement therapies, as is more commonly used in critically ill patients with renal failure. In these cases, the hemoadsorber may either be inserted before or after the hemofilter using the supplied tubing and where more rapid elimination of myoglobin, is required higher blood flows are often employed.

### The consensus process

To identify eligible studies, we conducted a literature search in PubMed (https://pubmed.ncbi.nlm.nih.gov) on 11th January 2023. Online conferences were held between 18th April − 4th September 2023 with the participation of all experts of the task force from the field of critical care and nephrologists practicing in critical care. During the first phase of the consensus, relevant domains to develop were identified recognising the knowledge gaps in the field. Following agreement, questions were developed (Table 1) and the Delphi process was used to achieve consensus using an online anonymous voting platform [38]. For defining the strength of the consensus, we used the following criteria:

#1 – Strong consensus: more than 90% agreement.

#2 – Consensus: 75 to 90% agreement.

#3 – Majority: 50 to 74% agreement.

#4 – No consensus: less than 50% agreement.

A second round of voting was conducted only in those cases defined by strength #2 (i.e.: 75 < and < 90% agreement). This process enabled us to test whether a reconsideration could result in a stronger consensus for these particular statements.

**Table 1:** Summary of guidance based on the consensus statements

Statements/questions		Responses (%)		Level of consensus
		Agree	Disagree	
Q1	Myoglobin can directly contribute to the development of AKI in rhabdomyolysis	100	0	Strong consensus - A
Q2*	Myoglobin can indirectly contribute to the development of AKI in rhabdomyolysis	100	0	Strong consensus - A
Q3	Myoglobin may also contribute to other organ dysfunctions than AKI in rhabdomyolysis	80	20	Consensus - A
Q4	Severe rhabdomyolysis should be considered when:			
	a) CK > 5,000 U/l	50	50	No consensus
	b) Myoglobin > 10,000 ng/ml	100	0	Strong consensus - A
	c) In the case of both parameters (CK and myoglobin) are available, myoglobin should be interpreted with priority	89	11	Consensus - A
	d) Criteria for RRT	50	50	No consensus
Q5	There is an association between high myoglobin/CK levels and the risk of the development of AKI	100	0	Strong consensus - A
Q6	Reducing the circulating level of myoglobin in severe rhabdomyolysis is beneficial in general	67	33	Majority - A
Q7	Reducing the circulating level of myoglobin in severe rhabdomyolysis might be beneficial for the kidneys	80	20	Consensus - A
Q8*	Reducing the circulating level of myoglobin in severe rhabdomyolysis is beneficial for the kidneys	80	20	Consensus - A
Q9*	Reducing the circulating level of myoglobin in severe rhabdomyolysis might be beneficial in general	93	7	Strong consensus - A
Q10*	Standard means of renal replacement therapy in the ICU do not significantly contribute to elimination of myoglobin.	87	13	Consensus - A
Q11*	HA can effectively remove circulating myoglobin	100	0	Strong consensus - A
Q12	HA can effectively remove circulating CK	72	28	Majority - A
Q13	HA can be considered as therapy for myoglobin removal in severe rhabdomyolysis	100	0	Strong consensus - A
Q14*	Therapy should ideally be started within 24 h after the onset of severe rhabdomyolysis	87	13	Consensus - A
Q15	Therapy should ideally be started within 24 h after the detection of severe rhabdomyolysis	67	33	Majority - A
Q16	Therapy should ideally be started within 12 h after the onset of severe rhabdomyolysis	67	13	Majority - A
Q17	Therapy should ideally be started within 12 h after the detection of severe rhabdomyolysis	67	13	Majority - A
Q18*	If the hospital is unable to measure myoglobin within a reasonably short timeframe, then treatment may be indicated based on the clinical picture and elevated CK levels.	93	7	Strong consensus - A
Q19	Consider CK > 5,000 U/l as criteria for diagnosis severe rhabdomyolysis in the absence of myoglobin.	56	44	Majority - A
Q20	HA can be used as a stand-alone hemoperfusion in a situation when CRRT is not required	67	33	Majority - A
Q21	HA can be used in combination with CRRT in patients who developed AKI and requiring CRRT	100	0	Strong consensus - A
Q22*	The HA cartridge should be changed after 8–12 h until achieving myoglobin values < 10,000 ng/ml, then at least every 24 h based on clinical response and values	87	13	Consensus - A
Q23	The optimal duration of hemoadsorption in severe rhabdomyolysis remains uncertain.	93	7	Strong consensus - A
Q24	The treatment with hemoadsorption in patients with AKI should be continued until CRRT is discontinued.	7	93	Strong consensus - DA
Q25*	In patients with AKI: The treatment should be continued until myoglobin values are < 5,000 ng/ml.	73	27	Majority - A
Q26	Do you consider defining absolute cut-off values for myoglobin and CK to start/stop therapy appropriate?	56	44	Majority - A
Q27	In patients without AKI: The treatment should be continued until sufficient myoglobin/CK reduction has been achieved and no AKI has developed.			
	a) Myoglobin < 10,000	44	56	Majority - DA
	b) Myoglobin < 5,000	56	44	Majority - A
	c) Myoglobin < 1,000*	27	73	Consensus - DA
	d) CK: <10,000	44	56	Majority - DA
	e) CK: <5,000*	40	60	Majority - DA
	f) CK: <1,000*	13	87	Consensus - DA
Q28	Testing rebound after interruption of HA of myoglobin/CK levels to determine continuation or discontinuation HA	93	7	Strong consensus - A
Q29*	In case of continued rhabdomyolysis or redistribution of myoglobin from other tissues into the blood stream, hemoadsorption treatment should be continued or re-installed.	87	13	Consensus - A

A, agreement; DA, disagreement; HA, hemoadsorption; CK, creatinine kinase; AKI, acute kidney injury; CRRT, continuous renal replacement therapy; *, questions required second round voting

## Results

The results are summarized in Table 1 together with the questions asked. There was generally good agreement allowing 6 consensus statements to be formulated.

*Consensus Statement 1*: Myoglobin contributes to the development of AKI through both direct and indirect mechanisms with raised myoglobin and CK levels associated with the risk of developing rhabdomyolysis (Strong consensus: Q: 1,2).

*Consensus Statement 2*: Patients with elevated myoglobin levels of > 10,000 ng/ml should be considered as a high-risk subgroup to develop severe secondary organ dysfunction, and myoglobin removal from the blood should be considered. Under such conditions reducing circulating levels may translate into a renal benefit also (Consensus Q: 3,5,6, 7, 8, 9).

*Consensus Statement 3*: Conventional renal replacement therapy does not significantly eliminate myoglobin whereas hemoadsorption can. As such hemadsorption should be considered for treatment of rhabdomyolysis and ideally started within 24 h in tandem with CRRT if this is also required (Consensus Q: 10, 11, 13, 14, 21).

*Consensus Statement 4*: If myoglobin cannot be measured then HA may be indicated based on both the clinical picture and CK levels (Consensus Q: 18, 19).

*Consensus Statement 5*: Although the optimal duration of HA therapy is unknown it was felt that HA cartridges should be replaced every 8–12 h until myoglobin levels < 10,000 ng/ml are achieved with continued measurement of myoglobin after treatment cessation. If myoglobin levels rebound HA treatment should be reinstated (Consensus Q: 22, 23, 28, 29).

*Consensus Statement 6*: In patients with AKI, HA can be discontinued before CRRT is terminated but should be maintained until the myoglobin concentration values are consistently < 5000 ng/ml (Consensus Q: 24, 25).

However, there were several areas where consensus could not be reached. These include the level of CK in isolation which could be used as a criterion for severe rhabdomyolysis although there was a majority agreement in terms of a value of > 5000 U/L being useful, particularly where myoglobin estimation is not readily available. Unsurprisingly, areas of contention focussed principally on issues regarding timing of hemoadsorption and relative triggers for commencing therapy. There was majority agreement that therapy should be started within 12 h after the onset of severe rhabdomyolysis and that stand alone hemoperfusion could be employed where CRRT was not required. With regard to stopping therapy in the absence of AKI, there was agreement that treatment should be continued with levels of myoglobin between 5 and 10,000 ng/ml though at levels below 1000 ng/ml treatment should be stopped. Similarly, CK levels < 1000 ng/ml should trigger cessation of therapy.

## Discussion

The role of myoglobin following primary injury and the subsequent cascade of events leading to rhabdomyolysis and AKI has been attributed to its effects on the proximal tubular epithelial cells. These include precipitation of myoglobin with Tamm-Horsfall protein leading to tubular cast formation and triggering of the inflammatory response with modulation of the NF-kB pathway as well as lipid peroxidation and cell death due to several mechanisms including ferroptosis [11, 39, 40]. Of note, most of rhabdomyolysis-associated tubular damage could be ascribed to myoglobin reabsorption through the endocytic receptor megalin located at the luminal cell surface [41]. Indeed, megalin interference and inhibition by using cilastatin limited rhabdomyolysis-induced AKI [42]. Continued elevation of myoglobin and perpetuation of the pathological injury correlates with prolongation of the anuric phase and delay of recovery of renal function. So far, although the rationale is strong, the results obtained with traditional extracorporeal purification methods have provided controversial data and therefore this has translated into a modest clinical impact.

However, blood purification techniques do have a role in current critical care practice. Although much attention has focussed on the use of blood purification in sepsis, these techniques have been shown to be successful in removing toxins and drugs, for example, removal of non-vitamin K antagonist oral anticoagulants in patients requiring surgery has been reported [43, 44]. Myoglobin removal, although still a controversial therapeutic goal due to a lack of solid data on its safety, feasibility and efficacy remains a potential therapeutic avenue in much the same way. Although high Cut-off filters have also been used to remove myoglobin these techniques are efficient only when very high fluid volumes are employed as in intermittent or extended haemodialysis, or hemodiafiltration but less so when used with conventional continuous techniques in the ICU. Furthermore, high volume techniques are rarely used in intensive care units and are often not available as an emergency therapy [19, 45, 46].

The available literature supports the fact that hemoadsorption seems to provide effective myoglobin and IL-6 removal, with effects on reduction of circulating CK levels, yielding a trend towards clinical improvement in these patients. Indeed, the most consistent finding in patients with rhabdomyolysis treated with hemoadsorption is the effective reduction in myoglobin levels. Hence the consensus reported here.

Similarly, CK is released to the bloodstream following the muscle cell injury and consequent release of phosphocreatine and transformation into creatinine. The serum levels increase following the rhabdomyolysis event, but this has not been associated with toxic effects [47]. Even though the detection of myoglobin in the serum is considered pathognomonic for rhabdomyolysis, CK is considered a more useful marker for diagnosis and severity assessment, due to its delayed clearance [48]. It is important to note, that rhabdomyolysis is commonly diagnosed by referring to specific CK cut-off values, the majority of which is a CK level > 1000 U/L or at least five times above the upper limit of normal reference values [49]. The consensus view is that there is promising, though insufficient evidence on the clinical outcomes associated with hemoadsorption therapy in rhabdomyolysis.

Several case reports have described recovery of renal function, expressed as progressive decrease of urea and creatinine values, increased urine output and/or termination of RRT [26, 28, 29, 31, 32, 34, 36, 37]. A recent retrospective propensity matched study including 95 patients suffering from severe rhabdomyolysis with myoglobin levels > 10,000 ng/ml and undergoing CRRT with (*n* = 55) or without (*n* = 40) CytoSorb^®^ therapy [49] found that kidney recovery occurred in a significantly higher proportion in the CytoSorb^®^ treated group (31.4 vs. 11.4%, *p* = 0.04). Larger case series included did not report data concerning this or other types of clinical outcomes. Hemoadsorption therapy itself does not seem to have any severe device related adverse events or complications related to its application. Mild thrombocytopenia after hemoadsorption therapy has been described in some case reports [29, 31], without symptoms or need for additional interventions.

Questions concerning the optimal criteria for starting therapy; which biochemical parameters and value cut-offs provide the best decision tools; the total myoglobin removal capacity by the adsorber; the monitoring of clinical outcomes and duration of therapy; remain unanswered. Also, larger prospective data or results from randomized trials are still not available, although 1 randomized trial (German clinical trial registry identifiers, https://drks.de:: DRKS00023998) as well as one prospective study (https://ClinicalTrials.gov Identifier: NCT04913298) are currently recruiting. These studies should help to fill the gaps in the currently available evidence and knowledge on the use of hemoadsorption therapy in this field.

## Conclusions

The results of the current consensus of the Hemoadsorption in Rhabdomyolysis Task Force support the view that adjuvant hemoadsorption therapy with CytoSorb^®^ is an effective method to reduce elevated circulating levels of myoglobin. However, the quality of the evidence is still low and therefore these results render the need for adequately designed clinical trials with clearly defined and relevant outcomes.

## Data Availability

No datasets were generated or analysed during the current study.
